# Identifying the Gaps in Human Papillomavirus (HPV) Vaccine Uptake: An Exploratory Factor Analysis of Adults in Tennessee

**DOI:** 10.3390/vaccines12121405

**Published:** 2024-12-12

**Authors:** Alina Cernasev, Oluwafemifola Oyedeji, Cary M. Springer, Tracy M. Hagemann, Kenneth C. Hohmeier, Kristina W. Kintziger

**Affiliations:** 1 Department of Clinical Pharmacy and Translational Science, College of Pharmacy, University of Tennessee Health Science Center, 301 S. Perimeter Park Dr., Suite 220, Nashville, TN 37211, USA; thageman@uthsc.edu (T.M.H.); khohmeie@uthsc.edu (K.C.H.); 2 Internal Medicine Residency Program, North Knoxville Medical Center, 7565 Dannaher Dr., Powell, TN 37849, USA; oluwafemifola.oyedeji@tennova.com; 3 Research Computing Support, Office of Innovative Technologies, The University of Tennessee, 2309 Kingston Pike, Suite 132, Knoxville, TN 37996, USA; springer@utk.edu; 4 Department of Environmental, Agricultural & Occupational Health, College of Public Health, University of Nebraska Medical Center, Omaha, NE 68198, USA; kkintziger@unmc.edu

**Keywords:** HPV, HPV vaccine, survey, adults, U.S.

## Abstract

**Background:** Human papillomavirus (HPV) remains the most prevalent sexually transmitted infection in the United States (U.S.). By the age of 45, over 80% of Americans will contract HPV, which creates a significant public health concern. Despite the availability of effective vaccines, low vaccination uptake continues to be a challenge, particularly in Tennessee. Additionally, the Advisory Committee on Immunization Practices (ACIP) recently expanded recommendations for HPV vaccine usage to include adults aged 27–45, suggesting a population with the potential to experience a gap in preventative care. To understand the underlying factors that may hinder Tennesseans from receiving the HPV vaccine, we conducted a cross-sectional survey from 29 June to 17 August 2023 among adults aged 18 to 45 in Tennessee. The survey was developed and informed by a scoping review regarding the various constructs and frameworks used in vaccine hesitancy and our previous qualitative work. Using theory-based instruments and previous qualitative data, this study aimed to determine the underlying factors that may hinder Tennesseans from receiving the HPV vaccine, focusing on those adults within the recently approved age range of 27–45 years old. **Methods:** An Exploratory Factor Analysis of 2011 participants ultimately included five factors, which explain 70.3% of the variability. These were Benefits/Trust, Perceived Susceptibility, Attitude/Behavioral Control, Perceived Barriers, and Perceived Severity. All Cronbach alphas were greater than 0.80, indicating that each factor was reliable. **Results:** When stratifying by various demographics, our analysis found that race emerged as a significant factor (*p* = 0.002), while the interaction of race and vaccination status was not significant (*p* = 0.753). Black respondents had significantly lower levels of Benefits/Trust than White (*p* < 0.001) and Asian respondents (*p* = 0.030), with no significant differences between White and Asian respondents. **Conclusions:** These findings underscore the importance of researchers, healthcare professionals, public health officials, and policymakers in addressing these demographic differences to effectively increase vaccination rates and reduce HPV-associated cancer risks in Tennessee. Further studies are needed for targeted interventions to address these disparities.

## 1. Introduction

Human papillomavirus (HPV) continues to be the most prevalent sexually transmitted infection in the United States (U.S.). Current reports from the Center for Disease Control and Prevention (CDC) suggest that the lifetime risk of infection is greater than 80% for women and 90% for men, with more than 80% of all Americans acquiring HPV by age 45 [1]. While most HPV infections are asymptomatic and will clear the body on their own, the consequences of HPV infection can be life-threatening. These negative outcomes include over 45,000 annual HPV-related cancer diagnoses in the U.S., including cervical, oropharyngeal, vulvar, vaginal, penile, and anal cancer, in addition to anogenital warts [1]. These outcomes pose a substantial financial burden of approximately USD 775 million in direct medical costs on the U.S. healthcare system [2].

HPV, a chronic sexually transmitted infection, is known to significantly impact the quality of life of affected patients, as evidenced by numerous studies [3,4]. For example, a study conducted in Iran reported changes in sexual function and quality of life (QoL) in women with HPV [5]. This study, while informative, also underscores the need for further research to fully understand the impact of sexual dysfunction associated with impaired sexual QoL [5].

To combat the spread of HPV and reduce morbidity and mortality, an effective strategy is proper utilization of the HPV vaccine. The first FDA approval of the HPV vaccine was for females in 2006, and approval for males followed soon after in 2011 [6]. Despite being included in the Healthy People 2020 and 2030 objectives, coverage and completion rates of the HPV vaccine have been historically low [7,8]. The suboptimal HPV vaccination coverage is more alarming in Tennessee. In 2020, the CDC reported that 1089 cancers were attributed to HPV in Tennessee [9]. Furthermore, a recent report from the Tennessee Immunization Information System showed that only 27.4% of adults aged 18 to 26 years in Tennessee were up to date with the vaccination series in 2023 [10].

The current guidelines from the Advisory Committee on Immunization Practices (ACIP) recommend catch-up HPV vaccination through the age of 26 for those who did not previously complete the series [11]. Furthermore, adults aged 27–45 years who are not adequately vaccinated against HPV may receive the HPV vaccine series based on shared clinical decision-making [11]. Despite the current ACIP recommendations, vaccination rates remain suboptimal in adults aged 27–45 years. Several studies have examined factors related to HPV vaccination in the adult population, which revealed barriers to vaccination such as healthcare accessibility, education level, race, and sex [12,13,14].

For example, people with an established healthcare provider are more likely to receive the HPV vaccine than those who do not have an established healthcare provider [15]. Another barrier to HPV vaccination is the level of education that one possesses. Those with a higher level of education are more likely to receive the vaccine. Additionally, although minority populations are disproportionately at a higher risk of morbidity and mortality attributed to HPV-related cancers, African American or Hispanic populations continue to have lower vaccination rates [12].

This study is the second part of a mixed-methods approach to understanding the perceptions, barriers, and facilitators of adults to receiving HPV and other recommended vaccines across diverse populations in Tennessee. The results from our qualitative components of this study have previously been published [16,17]. We used information obtained from a scoping review to develop a quantitative survey for adults between 18 and 45 years of age in Tennessee [18]. The objective of this survey was to determine the underlying factors that may hinder Tennesseans from receiving the HPV vaccine, focusing on those adults within the recently approved age range of 27–45 years old.

## 2. Methods

### 2.1. Study Design and Population of Interest

We have previously described detailed study methods, including participant recruitment and survey development and administration [16,17]. Briefly, from 29 June to 17 August 2023, we conducted a cross-sectional study that included an online self-administered survey to understand the barriers and facilitators of HPV vaccination. Our population of interest included Tennessee residents aged 18 to 45 years, with a specific focus on patients from 27 to 45 years old.

We developed the survey after an extensive review of the literature, extracting relevant survey items from published studies examining HPV vaccination across a variety of populations and using a variety of health behavior theories [18]. Survey development was further informed by previous qualitative work that used focus groups to understand barriers and facilitators to HPV vaccination among adults in Tennessee [16,17]. Our investigative team, which included experts in infectious diseases, epidemiology, pharmaceutical sciences, internal medicine, and health promotion and behavior, reviewed the entire set of questions. Additionally, results from validity and reliability testing for each question allowed researchers to make recommendations, including changes to wording, and missing items. A final set of proposed questions was sent to external reviewers, including experts in similar fields as our investigative team, for feedback. After incorporating this feedback, 31 survey items made up our final list of questions covering a variety of theoretical constructs across Theory of Planned Behavior, Social Cognitive Theory, and Health Belief Model, as well as investigator-developed items.

For survey questions relative to this analysis, we used 7-point Likert scales for responses to determine theoretical constructs that were positively and negatively associated with HPV vaccine measures. Possible responses ranged from “completely disagree” to “completely agree”. The questions included in this analysis were items representing specific theoretical constructs (i.e., Subjective Norms, Attitudes, Perceived Behavioral Control, Perceived Susceptibility, Perceived Severity, Perceived Barriers, Perceived Benefits, and Trust). We used a Qualtrics^XM^ (Provo, UT, USA) panel to ensure that the study sample represented our population of interest in terms of age, gender, race, ethnicity, religion, and residence.

### 2.2. Data Categorization

To ensure adequate representation of the sample, we incorporated questions related to age and ZIP code of residence to determine eligibility and other demographic characteristics. These variables included sex at birth, race, ethnicity, relationship status, education, and political and religious views. We recategorized the following variables based on data distribution, small sample sizes in certain categories, or needs for data analysis.

We stratified age as 18–28, 29–38, and 39–45 years. These age groups are of paramount importance in the context of HPV vaccination. They encompass adults who are or were eligible for the HPV vaccine, incorporating the expanded ACIP recommendations [11]. This underscores the critical role these age groups play in the vaccination landscape. The geographic region of Tennessee residents was categorized based on ZIP codes using the ZIP code approximation of the 2010 Rural-Urban Commuting Area (RUCA) codes [19]. RUCA codes were categorized as urban, suburban, large rural, and small rural areas. This comprehensive approach significantly enhances our understanding of the perceived barriers to accessing the HPV vaccine. Race/ethnicity was defined by self-selected survey categories: White, Black, Asian, and others (combined due to small cell sizes). For education level, the stratification was comprehensive, covering less than high school, high school graduate, some college, Associate/Bachelor degree, and Master/Doctoral/Professional degree. Study participants were asked questions about their sexual orientation, such as straight/heterosexual and other, and current relationship status, such as married/partnered/engaged and single/separated/divorced. Political leanings were defined by self-selected survey categories: liberal, moderate, and conservative. Study participants had the following options for employment: employed full or part-time, unemployed seeking/not seeking/disabled, and student.

### 2.3. Statistical Methods

We used Exploratory Factor Analysis (EFA) with an orthogonal (Varimax with Kaiser Normalization) rotation to improve the interpretability of our extracted latent constructs that influence HPV-vaccine decisions and uptake. We excluded one question on intention and one question related to Perceived Severity as they were only asked in either the vaccinated or unvaccinated groups, rather than both. The initial Factor Analysis included 29 questions pertaining to the seven theoretical constructs that were asked in both the vaccinated and unvaccinated samples. Factor loadings, which measure the relationship (i.e., strength and direction) of the item (i.e., variable) to the extracted factor, are reported. The final Factor Analysis included 25 items and the identified underlying factors. Two sets of hypothesized factors ended up loading together. Items for Benefits and Trust loaded into a single factor and items for attitudes and behavior control loaded together. We choose to report these five factors based on examining scree plots, factor loadings greater than 1, and comparing results of other EFA considering more or fewer factors on loadings and conceptual clarity. Four questions were removed based on this analysis, including three questions related to subjective norms due to greater variability within responses and one question related to trust due to concerns over interpretation of wording.

Survey item responses were given a value based on the 7-point Likert scale (e.g., 1 being “completely disagree” and 7 being “completely agree” depending on the directionality of the question). Survey items were averaged to create a mean score for each factor. That is, individuals’ responses to the survey items included in each factor were averaged to give every respondent a factor score, and then these scores were averaged across all respondents to provide a mean score for each factor. These averaged factor scores were used in the relational and comparative statistical analyses. We analyzed relationships between the factors using Pearson’s correlation coefficient (r).

Additionally, we used Multivariate Analysis of Variance (MANOVA) to determine differences in the five factors by demographic characteristics of interest. If the overall MANOVA tests were significant, indicating at least one of the factors differed by category of the demographic variable, we used individual Analysis of Variance (ANOVA) models with appropriate post hoc tests to determine which categories differed. Means and standard deviations (SD), overall MANOVA F-test *p*-value, and individual ANOVA F-test *p*-values were reported. We conducted all analyses in SPSS 29 (IBM Corp; Armonk, NY, USA) with a Type I error rate of 0.05.

## 3. Results

In this study, a total of 2011 participants provided complete surveys. The participants were more likely to be female (52.2%), White (81.2%), non-Hispanic (94.0%), aged 18–28 years (49.8%), with a high school diploma (37.4%), be politically moderate (37.2%), employed (68.0%), straight/heterosexual (77.9%), and single (56.0%). The majority of participants had not received a HPV vaccination (57.3%).

### 3.1. Exploratory Factor Analysis

The final EFA included 25 items that loaded on five factors, which explain 70.3% of the variability in HPV vaccine-seeking behaviors in adults. These factors included the following:

- Factor 1: Benefits/Trust;
- Factor 2: Perceived Susceptibility;
- Factor 3: Attitudes/Behavioral Control;
- Factor 4: Perceived Barriers;
- Factor 5: Perceived Severity.

Table 1 summarizes the survey items included in each factor and factor loadings. All factor loadings were 0.600 or higher, indicating strong correlations between the items and the factor. Table 2 contains the mean and standard deviation (SD) of each factor and the proportion of variance explained, as well as a measure of each factor’s internal reliability (Cronbach’s alpha). Factors 1 (Benefits/Trust) and 5 (Perceived Severity) had mean scores over 5, indicating individuals agreed with these factors. Factor 2 (Perceived Susceptibility) had a mean under 4, indicating disagreement. Each factor explained over 10% of the variability in HPV vaccine-seeking behaviors, with factor 1 (Benefits/Trust) explaining almost 20%. All Cronbach alphas were greater than 0.80, indicating that each factor was reliable. All factors were positively correlated with the others, except for Factor 4 (Perceived Barriers), which had a significant negative correlation with Factor 3 (r = −0.157, *p* < 0.001), a weak positive correlation with Factor 5 (r = 0.089, *p* < 0.001), and no significant correlation with the remaining factors (Table 3).

### 3.2. Latent Factors by Demographic Characteristics

Table 4, Table 5, Table 6 and Table 7 provide the results of the MANOVA and individual ANOVAs for significant associations with demographic characteristics of interest.

Vaccination Status. All factors, except Factor 4 (Perceived Barriers), were found to be significantly higher (all *p*-values < 0.001) for those who were vaccinated, including Benefits/Trust, Perceived Susceptibility, Attitudes/Behavioral Control, and Perceived Severity. Notably, Factor 4 was significantly higher for those not vaccinated (Table 4).

Age. The results of the MANOVA indicated that at least one factor had a significant age-by-vaccination status interaction (*p* = 0.004) and a significant main effect of age (*p* < 0.001). The individual ANOVAs played a crucial role in confirming the significance of age, revealing significant age-group differences for several factors (Table 5). For Factor 1 (Benefits/Trust), neither the main effect of age group (*p* = 0.112) nor the interaction of age group and vaccination status (*p* = 0.303) were significant.

Factor 2 (Perceived Susceptibility) had a significant age-by-vaccination status interaction (*p* = 0.002). To explore this interaction, age groups were compared within each vaccination status. Within the unvaccinated group, no age differences were identified (all *p*-values > 0.05). Within the vaccinated group, those aged 18–28 years old had significantly lower Perceived Susceptibility than those aged 39–45 years old (*p* = 0.007). No other significant differences were identified.

Factor 3 (Attitudes/Behavioral Control) revealed a significant main effect of age (*p* = 0.001), a finding of particular significance. The interaction of age group and vaccination status was not significant (*p* = 0.060). Pairwise comparisons were run to determine how age groups differed. Individuals aged 29–38 years old had significantly higher Attitude/Behavioral Control than individuals aged 18–28 years old (*p* = 0.036) and 39–45 years old (*p* = 0.002). No difference was found between 18–28- and 39–45-year age groups (*p* = 0.156). In Factor 4, it is worth noting that neither the main effect of age (*p* = 0.167) nor the interaction of age and vaccination status (*p* = 0.213) were significant.

Factor 5 (Perceived Severity) revealed a significant main effect of age (*p* < 0.001), a finding that significantly impacts the interpretation of the results. The interaction of age and vaccination status was not significant (*p* = 0.080). Pairwise comparisons showed that individuals aged 18–28 years old had significantly lower Perceived Severity than individuals 29–38 years old (*p* = 0.011) and 39–45 years old (*p* < 0.001). There was no difference between 28–38 and 39–45-year age groups (*p* = 0.362).

Sex at Birth. The MANOVA results indicated a significant effect of sex (*p* < 0.001) with at least one factor. Sex by vaccination status was not substantial (*p* = 0.414). To determine how sex differs by factor, individual ANOVAs were examined. Significant sex differences were found with Factor 2 (*p* = 0.029) and Factor 4 (*p* < 0.001). Males had significantly higher levels of Perceived Susceptibility, but considerably lower levels of Perceived Severity than females (Table 6).

Race. The analysis focused on three racial groups, White, Black, and Asian participants, due to limited sample sizes in other categories. The results of the MANOVA revealed a significant interaction between race and vaccination status (*p* = 0.026), as well as a significant main effect of race (*p* < 0.001) across at least one factor. Further examination through individual ANOVAs and post hoc analyses unveiled significant differences in perceptions related to vaccination across these racial groups (Table 7).

Factor 1 (Benefits/Trust) revealed significant findings. Race emerged as a significant factor (*p* = 0.002), while the interaction of race and vaccination status was not significant (*p* = 0.753). Black respondents had significantly lower levels of Benefits/Trust than White (*p* < 0.001) and Asian respondents (*p* = 0.030), with no significant differences between White and Asian respondents. For Factor 2 (Perceived Susceptibility), there was a significant race–vaccination status interaction (*p* = 0.006). To explore this interaction, race was compared within each vaccination status. Within the vaccinated group, no race differences were identified (all *p*-values > 0.05). Within the unvaccinated group, White respondents had significantly lower Perceived Susceptibility than Black (*p* < 0.001) and Asian respondents (*p* = 0.009). No significant differences were noted between Black and Asian respondents.

Factor 3 (Attitudes/Behavioral Control) had both significant overall (*p* < 0.001) and interaction effects between race and vaccination status (*p* = 0.039). In the unvaccinated group, Asian respondents had significantly higher Attitudes/Behavioral Control than both White (*p* = 0.001) and Black respondents (*p* < 0.001), with no significant differences between Asian and Black respondents. In the vaccinated group, Black respondents scored significantly lower on Attitudes/Behavioral Control compared to White (*p* < 0.001) and Asian respondents (*p* = 0.035). For Factor 4 (Perceived Barriers), neither the main effect of race (*p* = 0.820) nor the interaction of race and vaccination status (*p* = 0.402) were significant. For Factor 5 (Perceived Severity), the main effect of race was significant (*p* < 0.001), while the interaction of race and vaccination status was not (*p* = 0.993). Pairwise comparisons showed that Black respondents had significantly lower Perceived Severity scores than White (*p* < 0.001) and Asian respondents (*p* = 0.039), with no differences between White and Asian respondents (*p* = 0.688).

Location. No overall difference in factors was identified for location (*p* = 0.180), and the results are not reported.

## 4. Discussion

This study’s aim was to determine the underlying factors that may be associated with of the rate of uptake of the HPV vaccine among Tennesseans, focusing on those adults within the most recently approved age range of 27–45 years old. It further builds on previous work given that in our sample of 2011 online surveys, most patients were not vaccinated for HPV (57.3%), and were female, single, employed, straight/heterosexual, White, non-Hispanic, and between the ages of 18–28 years [20].

Our study’s findings demonstrated a significant increase (all *p*-values < 0.001) in Benefits/Trust, Perceived Susceptibility, Attitudes/Behavioral Control, and Perceived Severity among vaccinated people. It also underscores the potentially important role of healthcare providers in supporting and guiding patients through the vaccination process, making them an integral part of this decision. This could be useful information when designing future prospective studies or interventions to improve HPV vaccination rates in Tennessee or similar states. Additionally, this emphasizes the importance of improving education and communication around such a crucial topic. A study was conducted with minority women in the U.S. regarding the factors impacting the vaccination uptake in two age groups: 18–26 and 27–45 [21]. The findings revealed that the healthcare provider’s recommendation was the most influential aspect of receiving the HPV vaccine [21]. The study also highlighted other factors, including conversations about the benefits of receiving the vaccine with a doctor, having health insurance, and disclosing of sexual orientation to the healthcare providers [21]. Healthcare providers can encourage conversations with those who feel they are not open to discussing the benefits of and concerns with vaccination. It is important to note that a strong recommendation from a healthcare provider has a significant impact on HPV vaccine intention and uptake, as shown in previous studies [22,23]. Despite the positive attitudes of the surveyed respondents in these studies, administration rates remained low, especially in the Southern U.S.

To enhance vaccination rates among eligible adults residing in the Southern U.S, it is imperative that targeted programs and policies are developed. Interventions that explore the influence of various healthcare professionals might be needed to address vaccine hesitancy and motivational barriers, such as the belief that the HPV vaccine is unnecessary or unsafe. These possible interventions include educational campaigns when adults visit other healthcare professionals, such as dental offices. Many oropharyngeal cancers are HPV in origin and are screened for by the dentist. One study surveyed dentists, hygienists, and dental students’ attitudes toward the HPV vaccine, focusing on their willingness to administer it in office [24]. Most respondents, particularly students, expressed willingness to administer the HPV vaccine if legally permitted [24]. The findings suggest a potential for broader professional scope with HPV vaccine administration in dental settings as a potential avenue to highlight additional strategies to enhance vaccine uptake rates. To address this, future studies may explore targeted interventions for adults who visit dental offices.

While the currently available HPV vaccines have demonstrated effectiveness in prevention of HPV infections, research has pointed toward possibilities of treating various HPV-related conditions (REF) [25,26]. This may lead to a greater use of these vaccines, and enhance the need for targeted educational campaigns and outreach by healthcare providers, especially in these populations [27].

Our survey respondents showed that race emerged as a significant factor (*p* = 0.002), while the interaction of race and vaccination status was not significant (*p* = 0.753). Our findings demonstrated that Black respondents had significantly lower levels of Benefits/Trust than White (*p* < 0.001) and Asian respondents (*p* = 0.030), with no significant differences between White and Asian respondents. Our findings also echo previous conclusions of the literature regarding the level of Benefits/Trust. Walter et al. in 2020 sampled women residing in the Southern U.S. aged 18–32 who visited the emergency department [28]. The study reported that the White respondents were significantly more likely to be aware of the HPV vaccine compared to Black or African American respondents [28]. Regarding the vaccination rates in this sampled population, it was reported that White respondents are more likely to have received the HPV vaccine than Black or African American respondents [28]. These findings underscore the importance of our research in understanding and addressing disparities in vaccination awareness and uptake. Similarly, a recent study investigated the differences in awareness of the HPV vaccine among racial and ethnic minority groups in the U.S. [29]. The study used data from the 2018 Health Information National Trends Survey (HINTS) and found significant disparities in awareness levels [29]. According to the study, Asian Americans showed the lowest awareness of both HPV and the HPV vaccine compared to other racial and ethnic groups [30]. Future studies should use this evidence to inform the development of targeted interventions or adaptation of existing interventions to increase HPV vaccine intention and uptake in minority groups. This would ultimately reduce the burden of HPV-associated cancers.

Our study, which compares race with HPV vaccination status, has important implications. Within the vaccinated group, no race differences were identified (all *p*-values > 0.05). However, within the unvaccinated group, White respondents had significantly lower Perceived Susceptibility than Black (*p* < 0.001) and Asian respondents (*p* = 0.009). Previous research regarding race comparisons has shown that higher HPV knowledge scores were associated with vaccine series completion [30,31]. Those who perceived the vaccine as more beneficial were more likely to have completed the series [30]. Oh et al. (2021) reported that African American students were less likely to complete the series compared to non-Hispanic White students [32]. Further studies are needed for targeted interventions to address these disparities.

## 5. Limitations

We originally planned on conducting a secondary survey in this population, with the intent of performing confirmatory analyses. However, due to changes in university system contracts, the original survey’s deployment was significantly delayed. Based on our preliminary results and significant findings, we decided to postpone additional surveys to ensure the timely publication of this work.

Furthermore, due to the above challenges, two aspects of the survey design were overlooked, which unfortunately had the potential to impact study findings. This limited our ability to include one question on Perceived Severity and led to the incorrect categorization of age groups (for example, 18–28 and 29–38 instead of 18–26 and 27–36). However, based on the resulting data, this did not appear to significantly alter our results.

These findings are from a convenience sample of Tennesseans and may not represent the entire population of the state and might not be applicable to other states. However, our study included a large sample of respondents across Tennessee and was designed to match the demographic characteristics of the state in terms of age, gender, race, ethnicity, religion, and residence. The results indicate that our sample successfully matched the demographic characteristics of Tennessee, compared to U.S. Census data. Further, even within this limited focus, knowledge about HPV varied based on age, education level, and attitudes toward vaccination. States with similar demographic, cultural, and religious beliefs as Tennessee may identify similar patterns in constructs among vaccinated individuals.

Finally, surveys that rely on self-reported responses are subject to risk of recall bias or social desirability bias. We asked questions about HPV vaccination amidst a list of other standard vaccinations to decrease social desirability bias and increase the likelihood of recall. The survey was also conducted online, further reducing the risk of inaccurate reporting due to social stigma. Our sample included participants between the ages of 18 and 45 years, with oversampling in the 18–26-year range, so the sample is likely to have a more accurate recall of vaccination events that occurred recently. Further, we asked about vaccination history using targeted, structured questions that included prompts about offered versus received vaccines. We did not ask about dates of vaccination or number of doses. Our rate of vaccination in this sample (42.7%) is higher than the rate reported for Tennessee in 2022 (26.1% in 18–26-year-old participants) [33]. The state HPV vaccination rate is likely to be an underrepresentation as data do not include information on those over 26 years of age, and it only includes mandatory reporting of federally funded vaccines. All other vaccine reporting is voluntary in Tennessee [10].

## 6. Strengths

To our knowledge, our study is the first to explore barriers and facilitators to HPV vaccination in Tennessee and one of the first to specifically study these factors related to HPV vaccination in adults aged 27 to 45 years since the revised ACIP recommendations. The survey development was informed by the scoping review regarding the various constructs and frameworks used in vaccine hesitancy and the qualitative work. By partnering with an external company experienced in survey deployment and capturing representative survey samples, we were able to ensure that we had an adequate sample size in our exploratory survey deployment that was representative of our target population.

## 7. Conclusions

Our study found important associations between certain constructs or key concepts such as Benefits/Trust, Perceived Susceptibility, Attitudes/Behavioral Control, and Perceived Severity among vaccinated people. Such information is useful for informing future prospective studies or interventions. Our results demonstrate that healthcare practitioners must be cognizant of the existence of factors that hinder HPV vaccine uptake in Tennesseans. Most significantly, providers should consider the impact of sex and race, as these demographics are where most underlying disparities in this population exist. Attention to these characteristics and anticipation of vaccine hesitancy will also establish provider trust, which further influences the decision for many adults to get vaccinated. Awareness of these details will be useful in addressing additional obstacles that may arise for adults between 18 and 45 years who are contemplating HPV vaccination.

## Figures and Tables

**Table 1:** Factor loadings by survey item.

Survey Item ^1^	Factor Loading ^2^				
	1	2	3	4	5
Benefits 3. Getting vaccinated for HPV will decrease my chances of developing genital warts.	0.816				
Benefits 4. Getting vaccinated for HPV will decrease my chances of developing oral cancer.	0.783				
Benefits 2. If I get vaccinated for HPV, I can reduce my risk of cervical/penile or anal cancer.	0.776				
Benefits 1. Getting vaccinated for HPV will help protect me from genital HPV infection.	0.746				
Trust 1. I trust my healthcare provider’s decisions about which medical treatments are best for me.	0.728				
Trust 2. My healthcare provider is honest in telling me about all of the different treatment options available for my condition.	0.647				
Trust 4. All in all, I have complete trust in my healthcare provider.	0.629				
Susceptibility 4. If I do not get vaccinated for HPV, it is likely that I will develop a HPV-related cancer (e.g., oral, cervical, or penile) in the future.		0.863			
Susceptibility 2. If I do not get vaccinated for HPV and continue my current behaviors, it is likely I will become infected with genital HPV or develop genital warts in the future.		0.862			
Susceptibility 3. If I do not get vaccinated for HPV, I would feel vulnerable to developing a future genital HPV infection or genital warts sometime in the future.		0.839			
Susceptibility 5. If I do not get vaccinated for HPV, I would feel vulnerable to developing HPV-related cancer (e.g., oral, cervical, or penile) sometime in the future.		0.807			
Susceptibility 1. My current behaviors (e.g., sexual activity, drug use) put me at risk for HPV.		0.767			
Behavior Control 2. I am confident I can get all the recommended doses of the HPV vaccine in the next 12 months, even if my schedule is busy.			0.806		
Behavior Control 1. I am confident I can get all the recommended doses of the HPV vaccine in the next 12 months, even if there is a financial burden.			0.802		
Behavior Control 3. I am confident I can find a healthcare provider (for example, clinic, health center, physician’s office) where I can get all the recommended doses of the HPV vaccine in the next 12 months.			0.677		
Attitude 2. I think getting all the recommended doses of the HPV vaccine in the next 12 months is necessary.			0.651		
Attitude 3. I think getting all the recommended doses of the HPV vaccine in the next 12 months would be beneficial.			0.636		
Attitude 1. I think getting all the recommended doses of the HPV vaccine in the next 12 months would be protective.			0.600		
Barriers 2. Concerns about whether the vaccine is safe.				0.824	
Barriers 1. Concerns about possible side effects of the vaccine.				0.805	
Barriers 4. I do not have enough information about the HPV vaccine.				0.762	
Barriers 3. I do not have enough information about HPV.				0.755	
Severity 3. Having genital warts would have major consequences on my life.					0.822
Severity 1. Having cervical/penile or anal cancer due to HPV would have major consequences on my life.					0.821
Severity 2. Having cervical/penile or anal cancer due to HPV would be devastating for me.					0.818

^1^ Colors are used to highlight the distinct factors identified. ^2^ Extraction method: Principal Components Analysis. Rotation method: Varimax with Kaiser Normalization. Factor 1: Benefits/Trust, Factor 2: Perceived Susceptibility, Factor 3: Attitudes/Behavioral Control, Factor 4: Perceived Barriers, Factor 5: Perceived Severity.

**Table 2:** Factor summary statistics and internal reliability.

Factor	% Variance ^1^	Mean ^2^	SD ^3^	Cronbach’s Alpha
1. Benefits/Trust	19.675	5.04	1.51	0.908
2. Perceived Susceptibility	15.306	3.46	1.67	0.901
3. Attitude/Behavioral Control	14.761	4.77	1.49	0.908
4. Perceived Barriers	10.460	4.30	1.51	0.874
5. Perceived Severity	10.103	5.40	1.64	0.809

^1^ Percent of total variance explained by each factor. ^2^ Average of items within each factor. ^3^ Standard deviation of items within each factor.

**Table 3:** Correlations matrix of factors.

Factor	Factor 1	Factor 2	Factor 3	Factor 4	Factor 5
1: Benefits/Trust	1.000	0.285 *	0.655 *	0.017	0.569 *
2: Perceived Susceptibility	0.285 *	1.000	0.381 *	0.039	0.140 *
3: Attitudes/Behavioral Control	0.655 *	0.381 *	1.000	−0.157 *	0.491 *
4: Perceived Barriers	0.017	0.039	−0.157 *	1.000	0.089 *
5: Perceived Severity	0.569 *	0.140 *	0.491 *	0.089 *	1.000

* *p* < 0.001.

**Table 4:** Means of factors by vaccination status.

Factor	Vaccination Status				
	Unvaccinated (*n* = 1148)		Vaccinated (*n* = 855)		*p*-Value
	Mean ^1^	SD ^2^	Mean ^1^	SD ^2^	
1: Benefits/Trust	4.68	1.33	5.34	1.31	<0.001
2: Perceived Susceptibility	3.05	1.56	4.02	1.65	<0.001
3: Attitudes/Behavioral Control	4.25	1.38	5.49	1.33	<0.001
4: Perceived Barriers	4.52	1.48	4.00	1.49	<0.001
5: Perceived Severity	5.24	1.73	5.64	1.47	<0.001

^1^ Average of items within each factor. ^2^ Standard deviation of items within each factor.

**Table 5:** Means of factors by age.

Factor	Vaccination Status	Age	Mean ^1^	SD ^2^	N
1: Benefits/Trust ^3^	Combined	18–28 years old	4.9517	1.35621	996
		29–38 years old	4.9961	1.35540	504
		39–45 years old	4.9503	1.36615	503
2. Perceived Susceptibility	Unvaccinated	18–28 years old	3.1079	1.49845	492
		29–38 years old	3.1271	1.59065	277
		39–45 years old	2.9148	1.62159	379
	Vaccinated	18–28 years old ^5^	3.8989	1.62254	504
		29–38 years old	4.1075	1.59803	227
		39–45 years old	4.3855	1.77611	124
3: Attitudes/Behavioral Control ^4^	Combined	18–28 years old	4.7641	1.51152	996
		29–38 years old ^5,6^	4.9194	1.44115	504
		39–45 years old	4.6591	1.48350	503
4: Perceived Barriers ^3^	Combined	18–28 years old	4.9517	1.35621	996
		29–38 years old	4.9961	1.35540	504
		39–45 years old	4.9503	1.36615	503
5: Perceived Severity ^4^	Combined	18–28 years old ^5,7^	5.2711	1.65268	996
		29–38 years old	5.4964	1.60073	504
		39–45 years old	5.5891	1.62592	503

^1^ Average of items within each factor. ^2^ Standard deviation of items within each factor. ^3^ No significant effects found for main effect or interaction of main effect by vaccination status. ^4^ No significant interaction found for main effect by vaccination status, so only descriptive statistics by main effect reported. ^5^ Significant effects identified compared to 39–45-year-old participants (*p*-values < 0.05). ^6^ Significant effects identified compared to 18–28-year-old participants (*p*-values < 0.05). ^7^ Significant effects identified compared to 39–45-year-old participants (*p*-values < 0.05).

**Table 6:** Means of factors by sex at birth.

Factor	Sex				
	Male (*n* = 961)		Female (*n* = 1050)		*p*-Value
	Mean ^1^	SD ^2^	Mean ^1^	SD ^2^	
1: Benefits/Trust	4.963	1.973	5.049	1.839	0.153
2: Perceived Susceptibility	3.622	2.422	3.463	2.197	0.029
3: Attitudes/Behavioral Control	4.820	2.063	4.902	1.883	0.184
4: Perceived Barriers	4.306	2.242	4.236	2.063	0.300
5: Perceived Severity	5.291	2.242	5.554	2.242	<0.001

^1^ Average of items within each factor. ^2^ Standard deviation of items within each factor.

**Table 7:** Means of factors by race.

Factor	Vaccination Status	Race	Mean ^1^	SD ^2^	N
1: Benefits/Trust ^4^	Combined	White	5.0118	1.35846	1550
		Black ^5,6^	4.7487	1.32908	356
		Asian	5.2143	1.28407	42
2. Perceived Susceptibility	Unvaccinated	White ^6,7^	2.9334	1.58214	894
		Black	3.4201	1.33048	206
		Asian	3.7917	1.84672	24
	Vaccinated	White	4.0217	1.70414	656
		Black	3.9133	1.41088	150
		Asian	4.4333	1.31283	18
3: Attitudes/Behavioral Control	Unvaccinated	White	4.2463	1.38480	894
		Black	4.1362	1.26799	206
		Asian ^5,7^	5.1389	1.30001	24
	Vaccinated	White	5.5727	1.28349	656
		Black ^5,6^	5.1171	1.47651	150
		Asian	5.8241	1.19157	18
4: Perceived Barriers ^3^	Combined	White	4.3219	1.52378	1550
		Black	4.2835	1.42419	356
		Asian	4.2500	1.34005	42
5: Perceived Severity ^3^	Combined	White	5.5296	1.59973	1550
		Black ^5,6^	4.8876	1.70460	356
		Asian	5.4286	1.52181	42

^1^ Average of items within each factor. ^2^ Standard deviation of items within each factor. ^3^ No significant effects found for main effect or interaction of main effect by vaccination status, so only descriptive statistics by main effect reported. ^4^ No significant interaction found for main effect by vaccination status, so only descriptive statistics by main effect reported. ^5^ Significant effects identified compared to White participants (*p*-values < 0.05). ^6^ Significant effects identified compared to Asian participants (*p*-values < 0.05). ^7^ Significant effects identified compared to Black participants (*p*-values < 0.05).

## Data Availability

The original contributions presented in the study are included in the article.
